# Genome-wide identification and expression analysis of GDP-D-mannose pyrophosphorylase and KATANIN in *Corymbia citriodora*

**DOI:** 10.3389/fpls.2023.1308354

**Published:** 2023-12-18

**Authors:** Chubiao Wang, Jianzhong Luo, Wenliang He, Anying Huang, Wanhong Lu, Yan Lin, Yuduan Ou

**Affiliations:** ^1^ College of Coastal Agricultural Sciences, Guangdong Ocean University, Zhanjiang, China; ^2^ Research Institute of Fast-Growing Trees, Chinese Academy of Forestry, Zhanjiang, China

**Keywords:** KATANIN, GDP-D-mannose pyrophosphorylase, *Corymbia citriodora*, expression analysis, wood

## Abstract

The GDP-D-mannose pyrophosphorylase (*GMP*) and microtubule severing enzyme KATANIN (*KTN*) are crucial for wood formation. Although functional identification has been performed in *Arabidopsis*, few comprehensive studies have been conducted in forest trees. In this study, we discovered 8 *CcGMP* and 4 *CcKTN* genes by analyzing the whole genome sequence of *Corymbia citriodora*. The chromosomal location, genome synteny, phylogenetic relationship, protein domain, motif identification, gene structure, cis-acting regulatory elements, and protein-interaction of *CcGMP* and *CcKTN* were all investigated. *KTN* has just one pair of segmentally duplicated genes, while *GMP* has no duplication events. According to gene structure, two 5’ UTRs were identified in *CcGMP4*. Furthermore, there is no protein-interaction between KTN and GMP. Based on real-time PCR, the expression of most genes showed a positive connection with DBH diameters. In addition, the expression of *CcGMP4* and *CcKTN4* genes were greater in different size tree, indicating that these genes are important in secondary xylem production. Overall, this findings will enhance our comprehension of the intricacy of *CcGMP*&*CcKTN* across diverse DBHs and furnish valuable insights for future functional characterization of specific genes in *C. citriodora.*

## 1 Introduction

**Figure 1:**
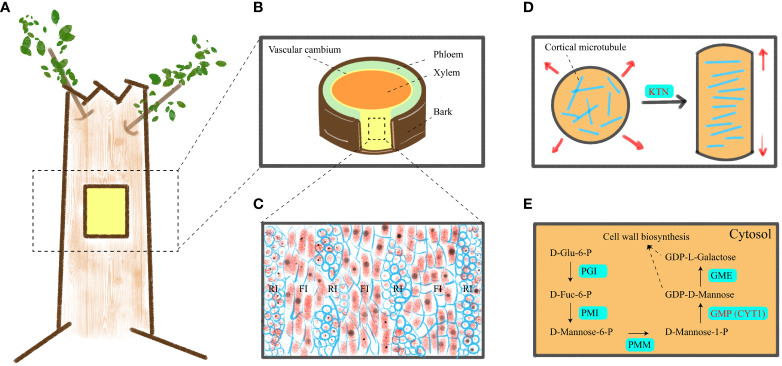
A schematic of vascular cambium, cell elongation mechanism and GDP-D-mannose synthesis. **(A)** Location of materials. **(B)** Transverse cutting of the stem. **(C)** Tangential longitudinal section of the cambium zone in a stem, revealing its anatomical structure. **(D)** Transverse cMT arrays support anisotropic elongation of plant cells. **(E)** Mechanism of GDP-D-mannose synthesis in cytoplasm. RI, FI, PGI, PMI, PMM, GMP, and GME indicated ray initials, fusiform initials, phosphoglucose isomerase, phosphomannose isomerase, phosphomannomutase, GDP-D-mannose pyrophosphorylase and GDP-D-mannose 3′, 5′ epimerase, respectively.

Wood (secondary xylem) originates from vascular cambium (**Figures 1A, B**), and its formation goes through a series of developmental processes, including secondary xylem mother cell differentiation, cell expansion, mass deposition of secondary walls and pit formation, programmed cell death, and heartwood formation (Plomion et al., 2001; Wang et al., 2021). The cambium consists of two meristem protocells, namely, fusiform initials and ray initials (**Figure 1C**). In angiosperms, fusiform initials mainly form parenchyma, ducts, and fibers, while ray initials produce rays, which mainly transport nutrients (Larson, 2012). At present, the genes related to vascular cambium in forest wood have been widely studied, including vascular cambium cell division and differentiation, cell wall synthesis (Ye and Zhong, 2015; Wang et al., 2021; Guo et al., 2022; Kim et al., 2022), etc. Therefore, given the role of vascular cambium in the secondary growth of trees, uncovering more genes related to vascular cambium is key to gaining insight into wood formation.

Wood consists of secondary cell walls and is composed mainly of three polymers: cellulose; Hemicellulose; and lignin. Cellulose consists of β-1, 4-linked chains of glucose units. It is synthesized by cellulose synthase complex (CSC) outside the plasma membrane. CSC moves within the plasma membrane and connects glucose subunits to each other to form cellulose microfibril. The movement path of CSC in the plasma membrane is mainly guided by cortical microtubules (cMT) (Paredez et al., 2006). Hemicellulose is composed of various chains, including glucomannan that links glucose and mannose residues via β-1,4 bonds (Lerouxel et al., 2006). Therefore, it can be seen that cMT and mannose play pivotal roles in wood formation (Mellerowicz and Sundberg, 2008).

Cytoskeleton and cell wall polysaccharides play an important physiological role in wood formation. Cortical microtubules control anisotropic cell expansion by directing the direction of cellulose microfibril (Baskin, 2001; Baskin, 2005). Transverse cMT arrangement causes cell growth (**Figure 1D**), while longitudinal cMT arrangement causes cell thickening (Kato et al., 2022). Furthermore, cMT are closely associated with the cell wall, and members of the cortical microtubule-associated protein family play a crucial role in regulating secondary cell wall synthesis during both secondary wall deposition and pit formation (Pesquet et al., 2010). The absence of genes encoding microtubule-associated proteins, such as *CORD1* (Cortical Microtubule Disordering 1), resulted in smaller pits within secondary cell walls (Sasaki et al., 2017). In *Arabidopsis*, *MOR1* (Microtubule organization 1), *ABS6* (Abnormal shoot 6), *SAV4* (Shade avoidance 4), *KTN*1 (Katanin 1), *PP2A* (Protein phosphatase 2A) genes are involved in the physiological activities of cMT (Li et al., 2021; Ren et al., 2022; Liu and Yu, 2023). However, these genes have been less studied in forest trees. Mannose is a constituent of the plant cell wall and is widely distributed in various plants. In the Smirnoff-Wheeler pathway (**Figure 1E**), GDP-D-mannose and L-galactose are utilized for cell wall metabolism, with GDP-D-mannose being synthesized by GDP-D-mannose pyrophosphorylase (GMP) (Lukowitz et al., 2001; Smirnoff et al., 2001). Additionally, GDP-D-mannose participates in ascorbic acid (Vitamin C) biosynthesis (Liu et al., 2022) and plays a crucial role in plant stress resistance. In previous studies, GMP activity is also affected by *KJC1* (KONJAC 1), *KJC2*, *CYT1* (Cytokinesis defective 1), *AMR1L1* (Ascorbate Mannose pathway Regulator 1 Like 1), *CSN5B* (COP9 signalosome subunit 5B), in which *CYT1* is closely related to cellulose synthesis (Lukowitz et al., 2001; Wang et al., 2013; Sawake et al., 2015; Ma et al., 2022). However, the mechanism of *KTN1* and *CYT1* genes in wood formation is still unclear, so we selected these two genes for in-depth study to identify whether they are related to wood formation.

*KTN1* is a heterodimer microtubule (MT) severing protein that uses energy from ATP hydrolysis to generate internal breaks along MT. Katanin p60 is one of the two subunits with ATPase and MT binding and severing activity, which is critical for cMT ordered and anisotropic cell expansion (Li et al., 2021), while the p80 subunit is responsible for targeting Katanin to certain subcellular sites (Burk et al., 2007). Microtubules are closely related to cellulose complex enzymes (Crowell et al., 2009; Lei et al., 2014), in which cellulose complex enzymes mainly control cell wall synthesis, while cMT are believed to alter lipid composition in the plasma membrane, thus affecting cellulose synthesis (Fujita et al., 2012). In addition, cMT also regulate the speed of cellulose synthase (Bischoff et al., 2011) and the movement track of cellulose synthase (Paredez et al., 2006). Microtubule severing protein Katanin can change the direction of cMT by cutting microtubules, thus indirectly affecting cell wall synthesis and changing cell morphology (Lindeboom et al., 2013). Among all individuals related to mutations in microtubule severing enzymes in *A. thaliana*, the majority exhibit cMT arrangement disorders, cell elongation defects, hypocotyl shortening, growth retardation and plant dwarfism (Burk et al., 2001; Webb et al., 2002; Bouquin et al., 2003). Notably, *ktn* mutants in *A. thaliana* have a significant impact on cellulose microfibrils (Burk et al., 2001), while overexpression of *KTN* leads to abnormal secondary walls (Zhou et al., 2007). The main economic component of forest is wood, and MT is closely related to the formation of wood (Pesquet and Lloyd, 2011). It can be seen that *KTN1* gene has certain physiological significance for wood formation.

*GMP1* (also known as *VTC1*、*CYT1*) is associated with mannose synthesis, encoding GDP-D-mannose pyrophosphorylase (Conklin et al., 1999), which is a key gene in the complex process of ascorbic acid (AsA) synthesis (Terzaghi and De Tullio, 2022). In *A. thaliana*, *cyt* mutants cause incomplete cell walls and excessive callose accumulation (Nickle and Meinke, 1998), especially the depletion of significant amounts of mannose from the cell wall (Lukowitz et al., 2001). Deletion of genes encoding GMP has also been found in rice, resulting in decreased mannose and galactose, as well as decreased cellulose synthase (Lamanchai et al., 2022). Overexpression of *GMP* gene has also been reported in various species, most of which are related to abiotic stress studies. It has been discovered that overexpression of *GMP* in *A. thaliana* compensates for the sensitivity of *myb30* and *myc2-2* mutants to submergence, and improves antioxidant biosynthesis (Yuan et al., 2017; Xie et al., 2023). Furthermore, overexpression of *GMP* increases AsA accumulation in rice, strawberry and soybean (Qin et al., 2016; Xue et al., 2018; Lin et al., 2021). Although *GMP* is highly expressed in the vascular tissues of trees (xylem is more highly expressed than phloem) (Nairn et al., 2008), the relationship between *GMP* gene and wood formation needs further study.

*Corymbia citriodora* exhibits rapid growth (de Araujo et al., 2021) and high wood density (Massuque et al., 2022), making it a widely cultivated species (Hung et al., 2016; de Souza et al., 2020). Its timber is also commercially valuable. In comparison to other *Eucalyptus* trees, *C. citriodora* can be utilized as premium-grade charcoal for steel production. (Couto et al., 2015; Massuque et al., 2022). Taken together, a larger diameter at breast height (DBH) of *C. citriodora* can bring more economic benefits. However, we found that trees of the same species and age showed different DBH, among which the number of *C. citriodora* with larger DBH was relatively small. Therefore, it is crucial to investigate whether the expression levels of *KTN* and *GMP* genes are associated with wood formation at different stages of DBH.

Nevertheless, no comprehensive investigation into the *GMP*&*KTN* genes of *C. citriodora* has been documented to date. However, with the unveiling of the *C. citriodora* genome (Healey et al., 2021), it is now possible to systematically scrutinize the potential functions of these genes in this species. In this study, 8 *CcGMP* and 4 *CcKTN* genes were identified from this species, two of which (*CcGMP4* and *CcKTN4*) are essential for wood formation. The results of our study will provide a basis for further functional identification of *CcGMP*&*CcKTN* genes in *C. citriodora*.

## 2 Methods

### 2.1 Identification of *GMP*&*KTN* genes in the *C. citriodora* genome

*C. citriodora* (Phytozome genome ID 507) and *A. thaliana* (447) gene sequence files, gene annotation files, protein sequences, and coding DNA sequence files were downloaded from the Phytozome (https://phytozome-next.jgi.doe.gov/) website. The protein sequences of *A. thaliana CYT1* (*At2g39770*) and *KTN1* (*At1g80350*) were compared with the *C. citriodora* genome to screen out candidate genes on the NCBI (https://blast.ncbi.nlm.nih.gov/Blast.cgi) website. Among them, all the selected *GMP* genes were subsequently identified, while the *KTN* genes were further artificially screened by using TBtools (Chen et al., 2020) to construct evolutionary trees. Putative *GMP*&*KTN* sequences were uploaded to Pfam (http://pfam.xfam.org/) and SMART (http://smart.embl-heidelberg.de/) databases to identify sequences protein domains (*AtCYT1*: PF00483, CL11394; *AtKTN1*: PF0004, CL38936). Due to the involvement of splice variants in the candidate genes, specific primers were designed by cutting a portion of the gene sequences based on the splicing pattern to distinguish different splice isoforms and analyze their expression levels. The protein sequences of *CcGMP*&*CcKTN* in the attached table (**Table S1**) can be referred to. Subsequently, the molecular weights (MWs), isoelectric points (pI), instability index (II), protein length and grand average of hydropathicity (GRAVY) of *CcGMP*&*CcKTN* proteins were calculated in the ExPasy website (https://web.expasy.org/protparam/). The subcellular location of *CcGMP*&*CcKTN* proteins was predicted by WoLF PSORT (https://www.genscript.com/wolf-psort.html?src=leftbar).

### 2.2 Chromosome localization and genome synteny analyses

Chromosomal locations of all *CcGMP*&*CcKTN* family genes were confirmed using the *C. citriodora* genome annotation file downloaded from Phytozome and mapped using the TBtools. *C. citriodora* gene sequence files were used to extract GC ratio and N ratio by Tbtools (parameters: window size is 10000; window overlap is 1000). The MCScanX software (Wang et al., 2012) (default parameters) was used to perform inter-species synteny analysis between *C. citriodora* and two representative plants, *A. thaliana* and *E. grandis*, as well as intra-species synteny analysis of *C. citriodora*. Homology relationships were also visualized by Tbtools software. Finally, the collinearity corresponding to *CcKTN*&*CcGMP* was highlighted.

### 2.3 Phylogenetic relationship and classification of GMP&KTNs

GMP&KTNs from *A. thaliana*, *O. sativa* and *E. grandis* were used for the classification of GMP&KTNs family in *C. citriodora*. These putative GMP&KTNs of other pattern plants were obtained by AtCYT1&AtKTN1 blast to their whole protein sequences. Subsequently, these putative protein sequences were submitted to the CDD (https://www.ncbi.nlm.nih.gov/Structure/bwrpsb/bwrpsb.cgi) for identification of the conserved domain (search for Pfam). ClustalW program (Thompson et al., 2002) (version 2.1; http://www.clustal.org/) was used for multiple sequence alignment, and TBtools was used to trim the data after sequence alignment. An unrooted maximum likelihood (ML) phylogenetic tree based on GMP&KTNs protein sequence was constructed by using the MEGA11.0 program (Tamura et al., 2021), with 1,000 bootstrap replicates under J+G+I+F model. The resulted ML tree was visualized in Chiplot (https://www.chiplot.online/tvbot.html). The subgroups were named following available information in *Arabidopsis* and rice (https://www.arabidopsis.org/; https://bis.zju.edu.cn/ricenetdb/).

### 2.4 Protein domain, motif identification and gene structure of *CcGMP*&*CcKTN*s

Conserved protein domains of the *CcGMP*&*CcKTN*s were blasted in the NCBI-CDD website (search for CDD). The *CcGMP*&*CcKTN* family gene structures were displayed by comparing the coding and genomic sequences with the TBtools. The obtained conserved motifs of the *CcGMP*&*CcKTN* family proteins were analyzed through the online website MEME (https://meme-suite.org/). Motif sequences were identified by InterPro online tool (https://www.ebi.ac.uk/interpro/).

### 2.5 Cis-element analysis for *CcGMP*&*CcKTN* gene promoters

The promoter sequences of 2,000 bp upstream of each *CcGMP*&*CcKTN* gene-coding region were extracted from the genome data. The PlantCARE online program (http://bioinformatics.psb.ugent.be/webtools/plantcare/html/) was used to search for assumed cis-acting elements. The cis-elements were annotated and visualized in a figure through the construction of a physical gene map using TBtools. The number of cis-elements associated with plant growth and development, phytohormone responsive, stress responsive and light responsive were also counted.

### 2.6 Protein-interaction analysis of CcGMP&CcKTNs

To reveal the structure and function of the CcGMP&CcKTNs protein interaction network, we performed protein interaction analysis based on known protein sequences from *E. grandis*. The protein sequences of CcGMP&CcKTNs were submitted to the STRING website (https://cn.string-db.org/) for analysis of protein interactions, and the predicted GO annotation results were visualized using R language.

### 2.7 Material acquisition, RNA isolation, qRT-PCR analysis and identification of differential genes

Cambium of 6.5-year-old *C. citriodora* with large (20.7–23.6 cm), medium (14.8–15.8 cm), and small (9.6–10.8 cm) diameters was collected at breast height (DBH) between 10 a.m. and 1 p.m. The DBH of different sizes were treated as three groups with six samples per treatment. All samples were collected and frozen in liquid nitrogen, then stored at −80 °C until used.

Total RNA was isolated using the Trelief RNAprep Pure Plant Plus Kit (Tsingke Biotech, Beijing China) according to the manufacturer’s instructions. Then, the RNA was used for first-strand cDNA synthesis with reverse transcriptase and the reverse transcription was performed using 1 mg of total RNA and PrimeScrip RT reagent Kit with gDNA Eraser (TaKaRa, Tokyo). Subsequently, cDNA was used as a template for qRT-PCR analysis using Premier 5.0 primers based on *C. citriodora* internal reference gene sequences (**Table S2**). The specificity of primers to their target genes was evaluated on the website EnsemblPlants (http://plants.ensembl.org/hordeum_vulgare/info/index). All of the primers were synthesized by TSINGKE Biotechnology Co., Ltd. (Beijing, China). A CFX96 real-time PCR system (Bio-RAD, Laboratories, Hercules, CA, USA) was used for qRT-PCR analysis. Each reaction mixture contained TransStart Top Green qPCR SuperMix 10 μL, cDNA template 1.5 μL, upstream primer 0.4 μL, downstream primer 0.4 μL, and sterile distilled water 7.7 μL. The reaction conditions were as follows: ① 94 °C, 30 s; ② 94 °C, 5 s; ③ 60 °C, 15 s; ④ 72 °C, 10 s. The analysis was completed after 40 cycles of reactions ②–④.

The relative expression levels of each treatment were calculated by the 2^−ΔΔCT^ method, and the mean ± standard deviation (SD) values were calculated from four independent biological replicates. The inner reference gene actin was utilized for data normalization, with *CcGMP7*&*CcKTN2* and the small DBH group serving as external controls. Differences in gene expression levels were analyzed via analysis of variance (ANOVA) followed by Student’s t-test in R software. After expression analysis, function verification of differentially expressed genes was performed based on GO annotation results.

## 3 Results

### 3.1 Identification of *CcGMP*&*CcKTN* genes

**Figure 2:**
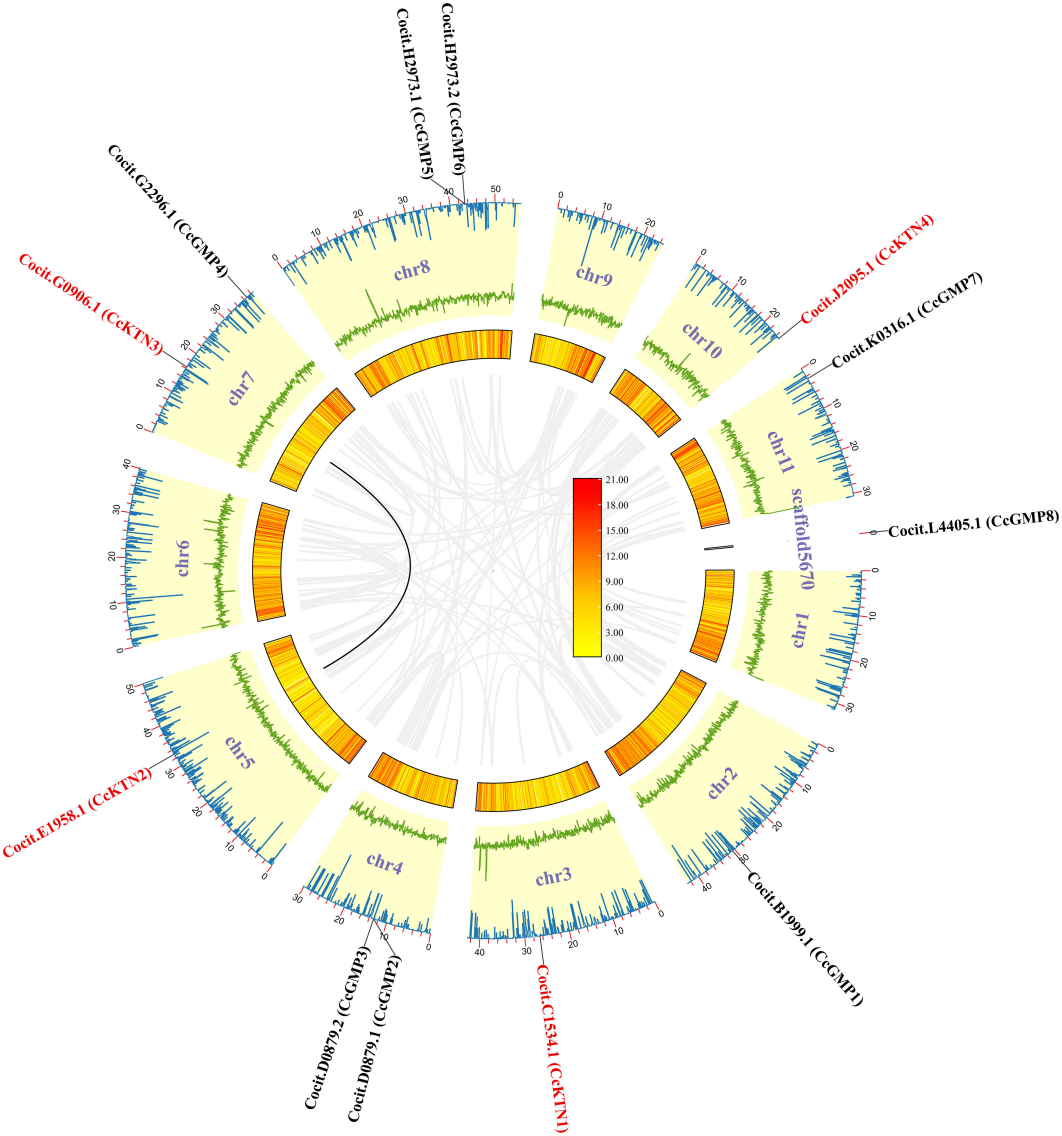
*CcGMP*&*CcKTN*s chromosomal localization and duplication events in the *C. citriodora* genome. From the inner to outer: gray line indicates all synteny blocks; black line highlights the collinear of *CcGMP*&*CcKTN*s pairs; heatmap for gene density profile; green line plot for GC ratio; blue line plot for N ratio.

This study used the protein sequences of *CYT1* and *KTN*1 genes in *A. thaliana* to identify homologous genes in *C. citriodora* using the BLAST method. After analyzing the conserved domains and removing redundant sequences, a total of 8 putative *CcGMP* genes and 4 *CcKTN* genes were identified, and the original splicing was manually trimmed. For convenience, we named the 8 *CcGMP* and 4 *CcKTN* genes based on their chromosomal location. The detailed information of all the studied genes was listed (**Figure 2**).

According to our analysis (**Table 1**), the predicted protein length, MW, pI, and IIV of the *CcGMP*s ranged from 257 to 726 AA, 28.31 to 81.62 kDa, 4.30 to 8.56, and 30.29 to 44.27, respectively. Additionally, *CcGMP4* exhibited the highest degree of homology to *AtCYT1* protein in the protein alignment. The predicted subcellular localization of *CcGMP*s (except for splice form *CcGMP3*, *6*) were in the cytoplasm. Furthermore, *CcGMP3*, *4*, and *7* are hydrophobic proteins, while the others are hydrophilic.

**Table 1:** Characteristics of *KTN*&*GMP* gene family in *C. citriodora*.

Gene ID	Gene name	Length (AA)	MW(Da)	pl	IIV	GRAVY	Subcellular location	Identity with query sequences (%)
Cci:Cocit.B1999.1.v2.1	*CcGMP1*	462	50,017.58	8.56	34.74	-0.013	Cytoplasm	22.34
Cci:Cocit.D0879.1.v2.1	*CcGMP2*	419	46,303.27	5.73	34.44	-0.007	Cytoplasm	30.07
Cci:Cocit.D0879.2.v2.1	*CcGMP3*	257	28,305.27	4.94	37.14	0.012	—	26.63
Cci:Cocit.G2296.1.v2.1	*CcGMP4*	361	39,513.33	6.77	30.29	0.109	Cytoplasm	91.14
Cci:Cocit.H2973.1.v2.1	*CcGMP5*	726	81,620.22	4.77	44.27	-0.279	Cytoplasm	26.00
Cci:Cocit.H2973.2.v2.1	*CcGMP6*	325	36,656.79	4.30	44.03	-0.420	—	—
Cci:Cocit.K0316.1.v2.1	*CcGMP7*	416	45,789.94	6.80	37.23	0.019	Cytoplasm	30.71
Cci:Cocit.L4405.1.v2.1	*CcGMP8*	305	33,509.59	6.26	31.37	-0.087	Cytoplasm	55.88
Cci:Cocit.C1534.1.v2.1	*CcKTN1*	658	72,937.52	8.82	51.49	-0.616	Chloroplast	43.14
Cci:Cocit.E1958.1.v2.1	*CcKTN2*	434	48,262.81	7.67	43.47	-0.521	Cytoplasm	44.21
Cci:Cocit.G0906.1.v2.1	*CcKTN3*	432	47,933.12	6.32	42.84	-0.544	Nucleus	41.62
Cci:Cocit.J2095.1.v2.1	*CcKTN4*	522	57,139.41	8.39	42.69	-0.561	Mitochondrion	85.45

AA, MW, pI, IIV, and GRAVY, respectively, indicate amino acid residues, molecular weight, theoretical isoelectric point, instability index values, and grand average of hydropathicity. CcGMPs were blasted to At2g39770 and CcKTNs were blasted to At1g80350. “—” means no results were available for subcellular localization prediction or sequence alignment data.

Moreover, we also predicted that the length of *CcKTN* proteins ranged from 432 to 658 AA, with a MW of 47.93 to 72.94 kDa and a pI of 6.32 to 8.82. The IIV range from 30.29 to 44.27, with *CcKTN4* being the most stable protein. Furthermore, *CcKTN4* showed the highest homology with *AtKTN*1. All 4 *CcKTN*s are hydrophilic. The subcellular localization prediction of *CcKTN* was not entirely reliable. *CcKTN1* may be localized in either chloroplasts or the nucleus, while *CcKTN3* is likely to be found in the nucleus and *CcKTN4* could potentially reside in either mitochondria or the cytoplasm. In general, microtubule severing regulation tends to occur within the cytoplasm, such as *AtKTN1*.

### 3.2 Chromosomal localization, and genome synteny of *GMP*&*KTN*s

To produce visual representations of the chromosomal positions of *CcGMP*&*CcKTN* genes, an analysis of the genomic distribution in chromosomes was conducted to determine their physical locations. This information was then used to generate the graphics (**Figure 2**). The result revealed that all *CcGMP*&*CcKTN*s, except for *CcGMP8*, were unevenly distributed on chromosomes in *C. citriodora*. Due to the important effect on functional differentiation and gene expansion, gene duplication events among *CcGMP*&*CcKTN*s were also investigated. In this study, one pair of segmental duplicated genes (*CcKTN2*/*3*) was revealed and distributed (**Figure 2**). To determine the selection constraints on the duplicated *CcKTN2*/*3*, we estimated the Ka/Ks ratio using the Tbtools and found that the value of the Ka/Ks ratio of *CcKTN2*/*3* was less than 1 (**Table S3**). These results indicated that these duplicated genes underwent strong purification/negative selection pressure, and almost no mutation occurred after duplication. To better understand the composition and structure of *C. citriodora* genome sequence, N ratio, GC ratio, and gene density were used for analysis. N ratio value indicated that the quality of *C. citriodora* genome sequence is poor and there may be missing, mismatched, or other abnormal sequences. GC content analysis results showed that the average GC content of every ten thousand bases is 0.4. Some segments of chromosomes 3, 8, and 6 have a higher GC content. Gene density distribution showed that most of the genes are concentrated at one or both ends of the chromosome.

**Figure 3:**
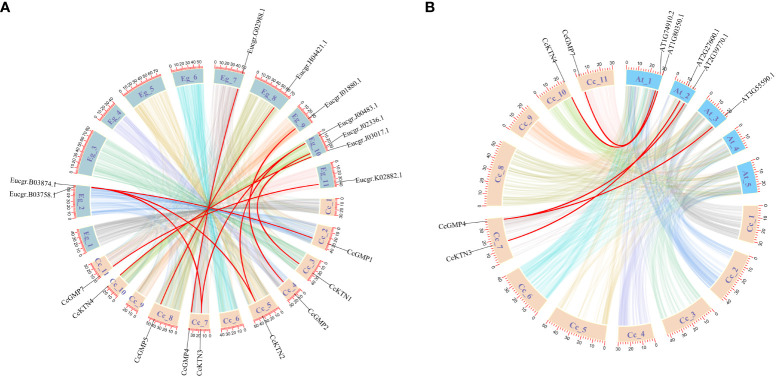
Synteny analysis of *CcGMP*&*CcKTN* genes between *C. citriodora* and two representative plant species (*A. thaliana* and *E. grandis*). **(A)** Syntenic relationship between *C. citriodora* and *Eucalyptus grandis*. **(B)** Syntenic relationship between *C. citriodora* and *Arabidopsis thaliana*. The colorful lines at the bottom indicate the collinear blocks within the *C. citriodora* and other plant genomes. The red lines indicate the pairs of *CcGMP*&*CcKTN* genes.

Based on a comparative micro-syntenic map of *C. citriodora* versus two representative plant species (including *A. thaliana* and *E. grandis*), we found 11 pairs of *GMP*&*KTN* orthologous genes between *C. citriodora* and *E. grandis*, and 5 pairs between *C. citriodora* and *A. thaliana* (**Figure 3**). According to information collected on the TAIR website (https://www.arabidopsis.org/), *CcGMP4*, *7* and *CcKTN4* may be involved in cellulose synthesis. Detailed genetic pairs are shown in **Table S4**.

### 3.3 Phylogenetic relationship and classification of *CcGMP*&*CcKTN*s

**Figure 4:**
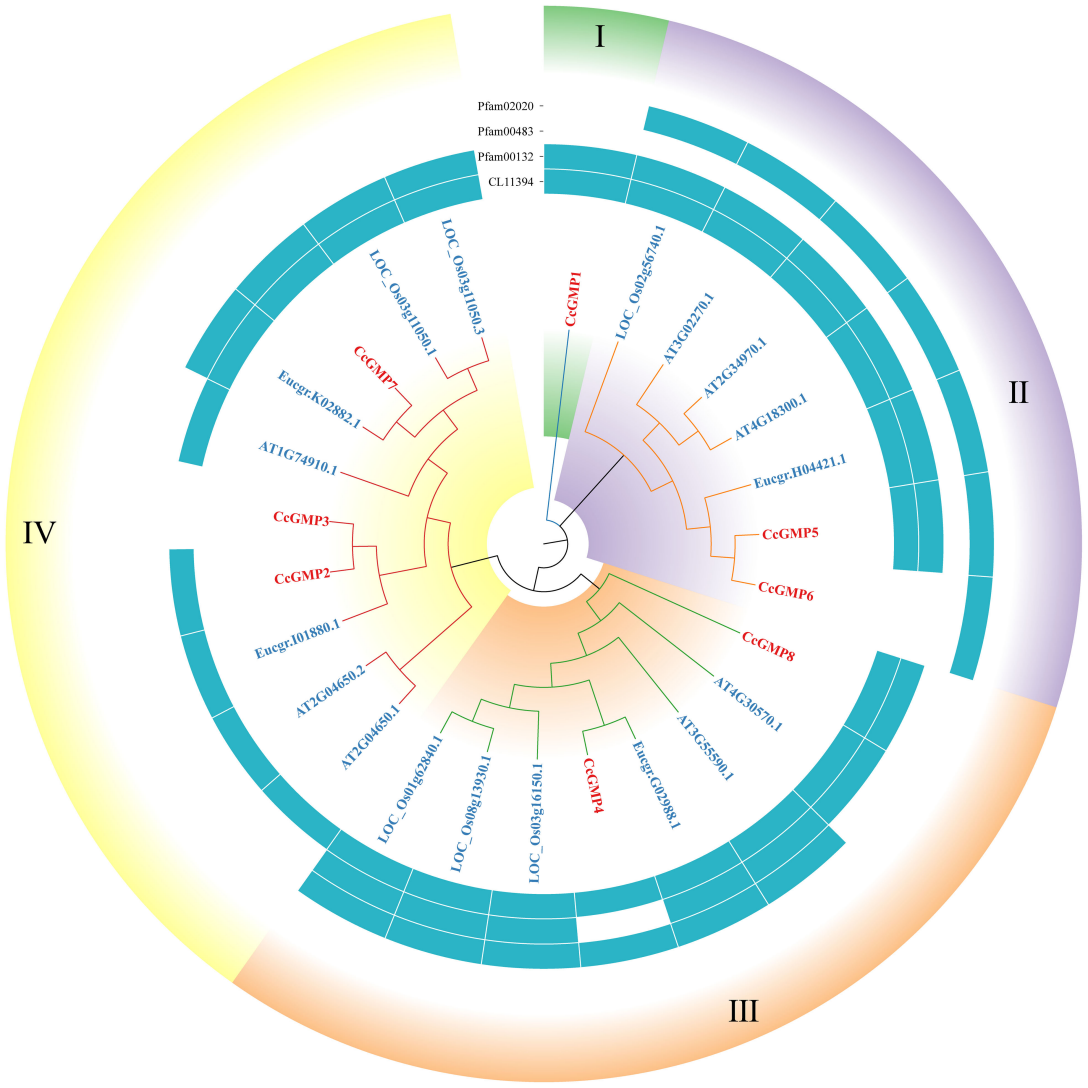
Phylogenetic tree of *GMP*s. Groups I-IV represent the classification of genes in the evolutionary tree. Conservative domains in the corresponding genes are represented by rectangular blocks in the color of teal.

**Figure 5:**
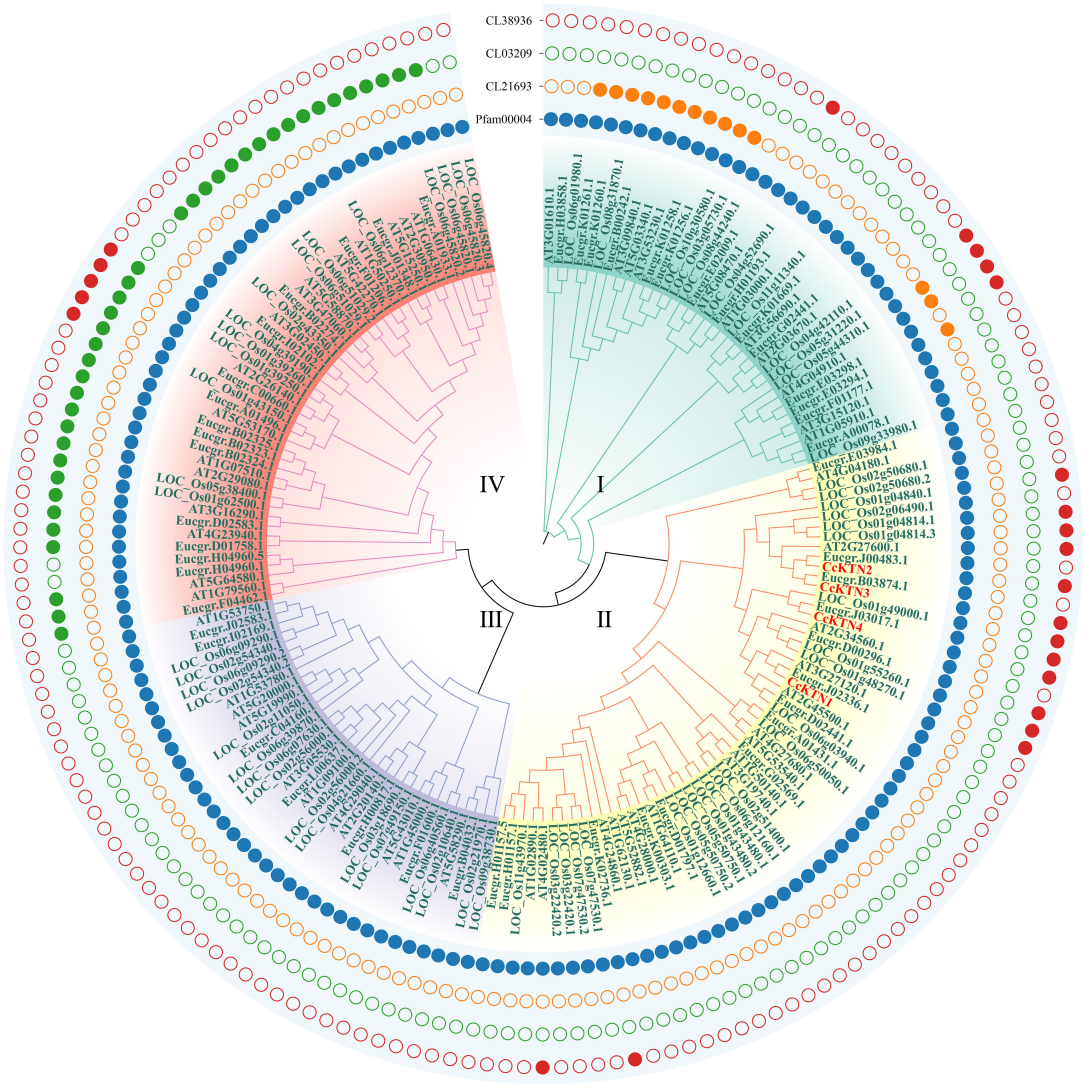
Phylogenetic tree of *KTN*s. Groups I-IV represent the classification of genes in the evolutionary tree. Solid and hollow circles correspond to the presence or absence of protein domains.

The protein sequences of GMP&KTNs from 4 species *O. sativa*, *A. thaliana*, *E. grandis*, and *C. citriodora* were used to investigate the phylogenetic relationship. An unrooted cladogram suggested that these *GMP* or *KTN*s were clustered into 4 categories and these categories contained members from both monocot and dicot plants except *GMP*-Group I (**Figures 4**, **5**). According to previous studies on *O. sativa* and *A. thaliana*, we can speculate the function of *GMP*&*KTN*s in *C. citriodora*.

Four conserved domains had been identified in *GMP*s (**Figure 4**), namely pfam02020 (W2 domain), pfam00483 (NTP transferase), pfam00132 (Hexapep transferase), and CL11394. Except for the splicing forms *CcGMP3* and *6*, all other genes shared the CL11394 domain that involved in Glycosyltransferases (GTs). The group II of *GMP*s contained pfam02020, which mainly mediates RNA transcription and processing. *Arabidopsis* genes within this group may play a role in root development and floral organ differentiation. In contrast, *Arabidopsis* genes belonging to groups III and IV lack the W2 domain but are involved in regulating AsA and mannose formation. The group III of genes mostly contains the conserved domain pfam00483, which mainly transfers nucleotides onto phosphor sugars, and rice genes involved in this group regulate the synthesis of mannose-1-phosphate guanylyltransferase (MPG). Therefore, based on the similarity of sequence and domain, we classified *CcGMP*s into four groups and postulated that *CcGMP2*, *4*, *7* and *8* are implicated in cell wall synthesis.

In addition, we also classified the genes related to *KTN* (**Figure 5**). All *KTN*s contained pfam00004 (ATPase family associated with various cellular activities, AAA), which related to performing chaperone-like functions. Only some genes in group I had cl21693 (Cell division cycle protein, CDC). Except group III some genes of the other groups contained cl38936 domain (P-loop_NTPase Superfamily), which is involved in diverse cellular functions. Most of the genes in group IV contain the cl03209 domain (Peptidase_M41 Superfamily), which may influence metallopeptidase activity. According to the clustering results, *CcKTN*s were all grouped into one category. In order to more accurately infer which gene was involved in cell wall synthesis, we verified by sequence similarity with *Arabidopsis*. According to the TAIR website, *A*t*KTN1* and *At2G34560* are involved in cell wall biosynthesis, so *CcKTN4* may participate in the same process.

### 3.4 Protein domain, gene structure, and motif identification of *CcGMP*&*CcKTN*s

**Figure 6:**
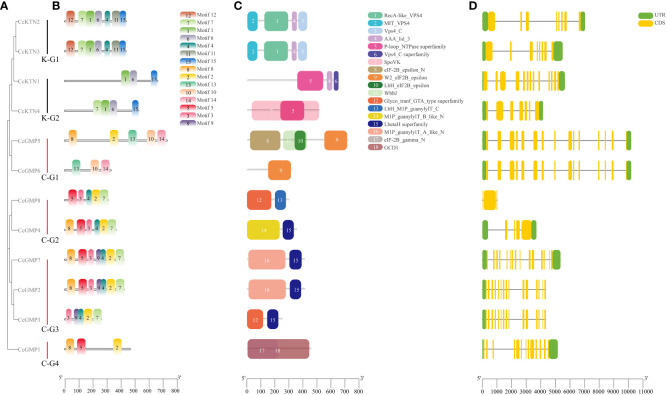
Motif, protein domain and gene structure of *CcGMP*&*CcKTN*s. **(A)** Phylogenetic relationship of the *CcGMP*&*CcKTN* gene family. *CcKTN* gene was divided into two groups and the *CcGMP* gene into four groups. **(B)** Protein motif composition of *CcGMP*&*CcKTN*s. **(C)** Conserved protein domains of *CcGMP*&*CcKTN*s. The domains and motifs were boxed in different colors. **(D)** Exon-intron structure of *CcGMP*&*CcKTN*s. CDS, UTR and intron are represented by yellow, green boxes and black lines, respectively.

The evolutionary relationship of *CcKTN*&*CcGMP*s was investigated by constructing a phylogenetic tree based on their amino acid sequences. CcKTN&CcGMP proteins were respectively classified into 2 and 4 subfamilies in this phylogenetic tree, which is basically consistent with the results shown in **Figure 6A** based on phylogenetic analysis of the 4 plant species.

To further investigate the diversity of the *CcKTN*&*CcGMP* genes, we analyzed their protein motifs using the MEME online server. Fifteen conserved motifs were identified, i.e., motifs 1 to 15 (**Figure 6B**). A detailed information of these protein motifs was presented in **Table S5**. Among the 12 gene products, *CcKTN1* and *CcGMP1*, *5*, and *6* lacked motif7. *CcKTN* genes shared the same motif1, 6, and 15. Except for *CcGMP6*, all genes of *CcGMP* shared the same motif2. In the InterPro website results, Motif3 and motif5 are potentially associated with nucleotide-diphospho-sugar transferases that participate in cell wall synthesis and these motifs are commonly found in *CcGMP*s, except for *CcGMP5* and *CcGMP6*.

The conserved domains of *CcKTN*&*CcGMP*s were investigated (**Figure 6C**). K-G1 shares the same domain, where domains1, 2, and 3 belong to the vacuolar protein sorting-associated protein 4 (VPS4) family. The VPS4 protein family is responsible for the decomposition and recycling of plasma membrane proteins in cells and plays an important role in cell metabolism and homeostasis maintenance. Domain4 plays an important role in the function of ATPase and can influence ATP hydrolysis and energy release by regulating the conformation of ATPase. K-G2 also has the same domain4. The presence of domain5 makes proteins usually have one or more nucleotide binding sites, which can bind to ATP, GTP, CTP, UTP and other nucleotides and release energy through hydrolysis of ATP or GTP. Notably, the SpoVK (The stage V sporulation protein K) domain in *CcKTN4* may be involved in cell wall synthesis.

There are three eukaryotic translation initiation factor (eIF) 2B epsilon subunit related domains in C-G1. W2_eIF2B_epsilon domain mainly interacts with eIF2B alpha, beta and gamma subunits to form a complete eIF2B complex. The eIF-2B_epsilon_N interacts with other proteins to regulate the function of eIF2B epsilon subunits. Lbh_eIF2B_epsilon is mainly involved in the formation and translation of eIF2B complex. Therefore, *CcGMP5*, *6* may be involved in translation regulation. Both C-G2 and C-G3 (except *CcGMP8*) genes shared the domain of LbetaH Superfamily, which plays important roles in a variety of biological processes, such as lipid metabolism, cell signal transduction, gene transcription and cell proliferation. Both C-G2 and C-G3 (excluding *CcGMP3*) are present in the (mannose-1-phosphate) M1P family, which is involved in the synthesis of GMP that plays a crucial role in maintaining cell wall integrity, morphogenesis and viability. Moreover, the GCD1 domain of C-G4 facilitates the synthesis of NDP-sugar pyrophosphorylase which contributes to cell wall synthesis.

To provide more valuable information on *CcKTN*&*CcGMP* genes, the gene structure is shown in **Figure 6D**. Exon numbers vary from 3 to 15, with *CcGMP2*, *3*, *5*, *6*, and *7* genes having the highest number of exons. Apart from the spliced copies *CcGMP3* and *CcGMP6*, different *CcGMP* genes ranged in length from 1,025 to 10,192 bp, while different *CcKTN* genes ranged in length from 3,759 to 4,351 bp. We found that the absence of 5’ and 3’ untranslated regions (UTR) in the *CcGMP8* gene may be caused by incomplete annotation information of the gene, or the mRNA produced by the gene may start directly from the start codon. Also, two 5’ UTRs were identified in *CcGMP4*. While some genes exhibit multiple 5’ UTRs, their translational efficiency, stability and other characteristics are likely to be impacted. Further investigation is required to determine whether these abnormalities affect gene expression.

### 3.5 Cis-element analysis of *CcGMP*&*CcKTN* genes in *C. citriodora*

To understand the genetic functions and regulatory mechanisms of *CcGMP*&*CcKTN*s, the majority of cis-elements in their promoter regions were analyzed. The 2,000 bp sequence upstream of *CcGMP*&*CcKTN*s was obtained as the putative promoter. The PlantCARE tool was utilized to conduct a scan of the promoter region of *CcGMP*&*CcKTN*s in search of potential shared cis-elements. Then, to more clearly express the specificity of some genes, we categorized cis-elements based on their functions related to plant growth and development, phytohormone response, stress response, and light response.

**Figure 7:**
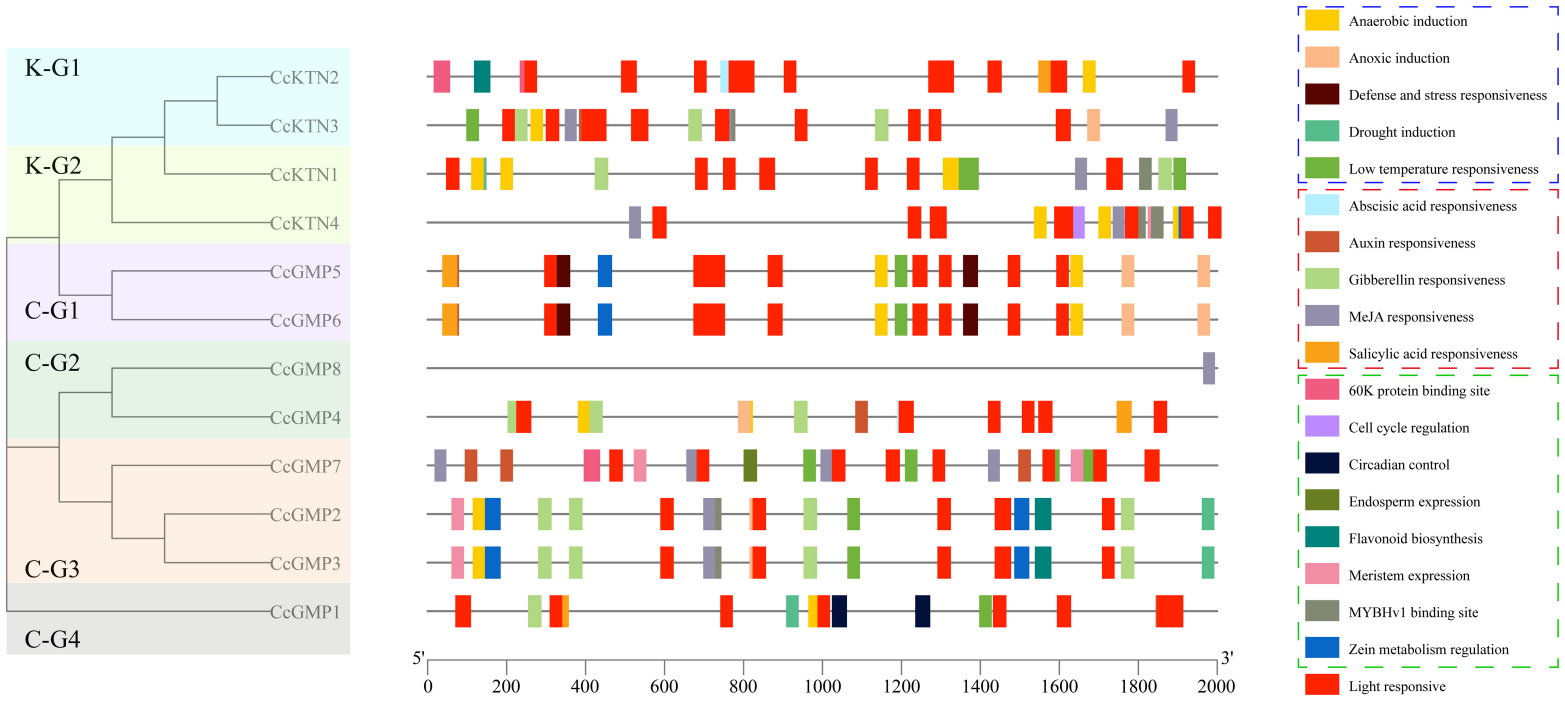
Cis-regulatory elements within the 2,000 bp upstream regions of putative *CcGMP*&*CcKTN* genes, arranged by the phylogenetic tree. Cis-elements involved in stress response, phytohormone response, and plant growth and development are represented by dotted boxes of blue, red, and green, respectively.

The number of cis-elements of different genes is detailed in **Table S6**. A total of 259 cis-elements were identified, of which 99 were photo-responsive, followed by 78 were related to plant hormone regulation. According to the different functions of each cis-element, we screened them and made drawings with TBtools. The results showed that the cis-elements regulating the light response were distributed in all genes (**Figure 7**), with the largest number of 14 in *CcKTN3*. It can be observed that the distribution of functional cis-elements in *CcGMP8* is limited, which may be attributed to incomplete gene annotation.

We delved deeper into the distinctiveness of each gene. Apart from light response and stress elements, only *CcGMP1* gene was implicated in physiological control. Only *CcGMP7* was involved in endosperm specific expression and possessed a 60K protein binding site. This implied that *CcGMP7* plays a role in the plant’s immune response, facilitating its defense against viral infections.

Among *CcKTN* genes, only *CcKTN4* was involved in meristem expression, cell cycle regulation and zein metabolism regulation. *CcKTN2* was solely involved in flavonoid biosynthesis and harbored the 60K protein binding site. Therefore, except for *CcGMP8*, most genes may exert an influence on growth.

### 3.6 Protein-interaction analysis of *CcGMP&CcKTN* genes in *C. citriodora*

With the exception of CcGMP2 and CcGMP8, all other proteins within the CcGMPs were found to interact with one another, as depicted in the **Figure S1**. Notably, CcGMP1 displayed robust reliability in interacting with CcGMP5 and CcGMP6, while CcGMP4 had strong interaction reliability with CcGMP3 and CcGMP7. In addition, two *E. grandis* homologous genes were introduced to link the relationships of CcGMP1, 5, 6 and CcGMP3, 4, 7. These two *Eucalyptus* genes had strong reliability in interacting with CcGMP1, 5, 6 and both *Eucalyptus* genes were involved in eIF-related functions. In the CcKTN proteins, CcKTN1, 2 and 3 exhibited mutual interaction, whereas only a low level of reliability is observed in the interaction between CcKTN4 and CcKTN1. In addition, we found no interaction between GMP and KTN proteins.

### 3.7 Expression patterns of *CcGMP*&*CcKTN* genes in different DBH

**Figure 8:**
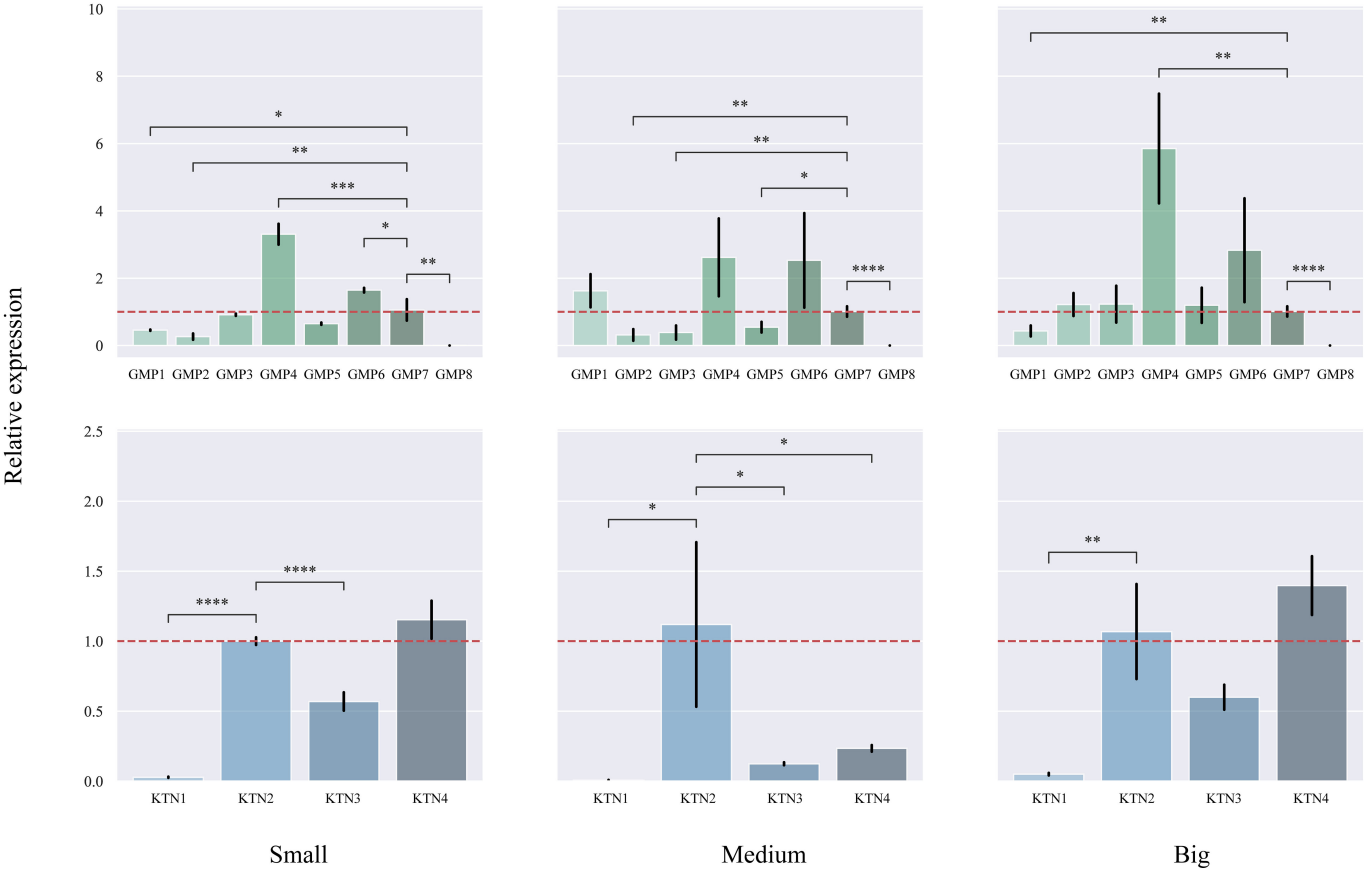
Quantitative real-time PCR (qRT-PCR) analyses in different genes. The *CcGMP*&*CcKTN* gene family was expressed in small, medium, and large DBH, respectively. Each treatment was controlled by *CcGMP7*&*CcKTN2* (relative expression level was close to 1). The two red dotted lines are located at 1 and −1 on the Y-axis. Error bars indicate the standard deviation of four biological replicates. *p ≤ 0.05, **p ≤ 0.01, ***p ≤ 0.001, ****p ≤ 0.0001.

We studied different sizes of DBH separately to find differentially expressed genes (**Figure 8**). In small DBH, the expression levels of *CcGMP4* and *CcGMP6* genes were significantly higher than those of reference genes. The expression of *CcKTN4* was higher than that of the reference gene, but the difference was not significant. In medium DBH, the expression levels of *CcGMP4* and *CcGMP6* genes were higher than those of reference genes, but the difference was not significant, and the expression levels of *CcKTN* genes were significantly lower than those of reference genes. In large DBH, *CcGMP4* was significantly larger than the reference gene, and *CcKTN4* was larger than the reference gene, but the difference was not significant. There was a large difference in the mean value of data between *CcGMP6* and *CcGMP7*, but no significant difference in the T-test results between the two groups, which may be due to too little biological duplication, resulting in a large variance. Therefore, *CcGMP4*, *CcGMP6* and *CcKTN4* can be regarded as differential genes for further analysis.

**Figure 9:**
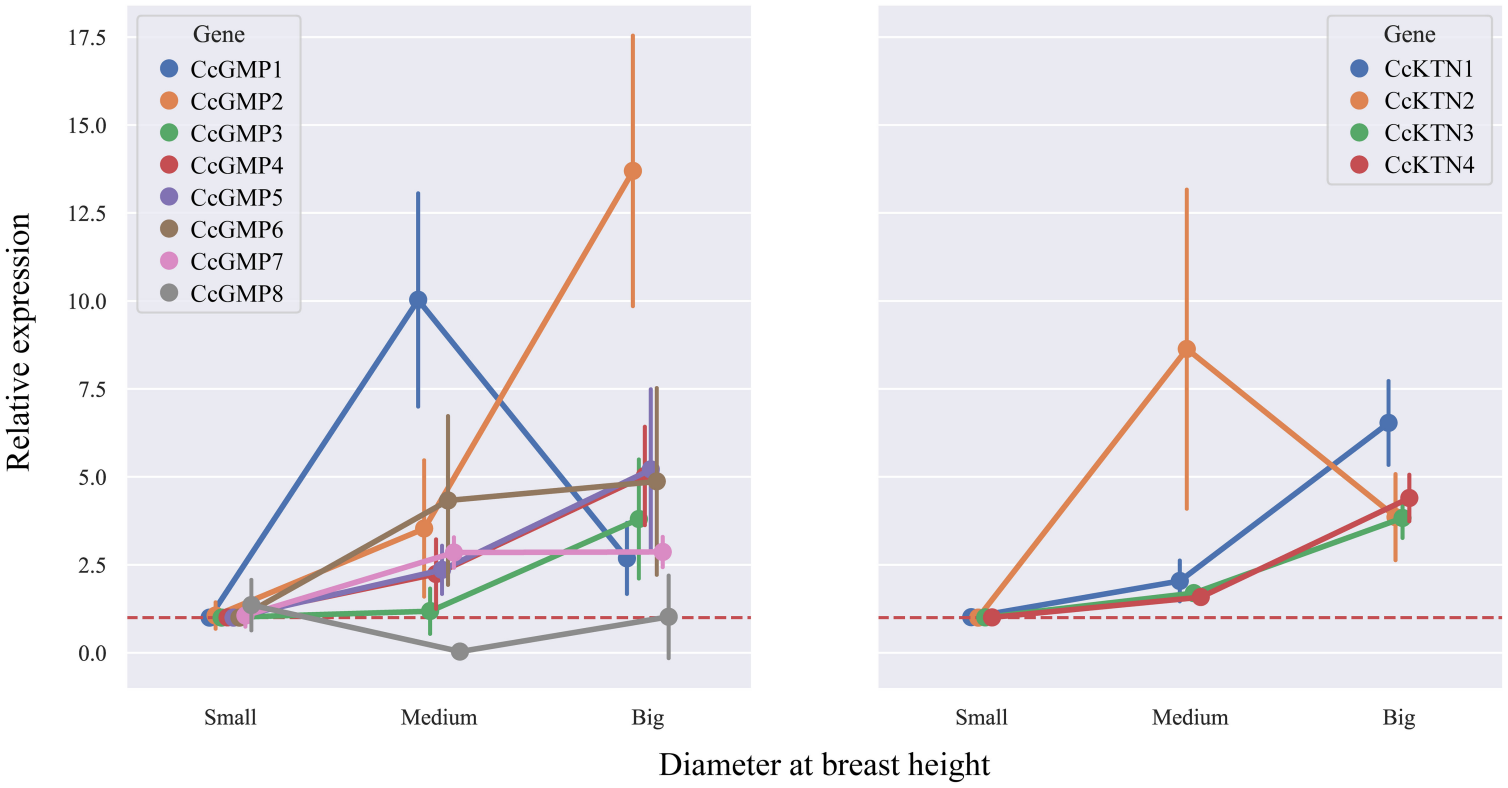
The relative expression in different DBH of *CcGMPs* and *CcKTNs*. Each gene was controlled by a small DBH (expression level close to 1). Error bars indicate standard deviation of four biological replicates.

In order to investigate the correlation between DBH and gene expression, we analyzed the changes in different genes based on varying DBH values (**Figure 9**). Among the *CcGMP* genes, most exhibited an upward trend with increasing DBH, particularly *CcGMP2*. Additionally, while the expression of *CcGMP1* was highest in trees with medium DBH, it decreased in those with larger diameters. With regards to *CcKTN* genes, all except *CcKTN2* showed a similar increase. Notably, the expression level of *CcKTN2* was extremely unstable at medium DBH, while the expression of other *CcKTN* genes was relatively stable. Therefore, further discussion is required to determine whether this variability is attributed to the characteristics of the *CcKTN2* gene itself or the biological processes involved.

### 3.8 Go annotation of *CcGMP*&*CcKTN* genes

From the expression results, differentially expressed genes (*CcGMP4*, *CcGMP6*, *CcKTN4*), abnormal genes (*CcGMP1*, *CcKTN2*) and reference genes (*CcGMP7*, *CcKTN2*) were selected for function prediction. Cellular component of these genes was located in cytoplasm (**Table S7**). Among the *CcGMP1*, *4*, *6*, and *7* genes, all the genes exhibited the molecular function of nucleotide transferase activity and participated in the biological process of nitrogen compound synthesis and organic compound synthesis in cells (**Figure S2A**). Except *CcGMP7*, all of them were involved in the molecular functions of organic and heterocyclic compounds. The genes *CcGMP4* and *CcGMP7* were involved in the biological process of mannose synthesis; *CcGMP1* and *CcGMP6* were active as guanylate exchange factors; *CcGMP1* and *CcGMP6* were involved in cytoplasmic transcription initiation factors; and *CcGMP1* were involved in cell development and immune cell conduction. It is evident that the expression level of *CcGMP1* is excessively high in medium DBH and low in large DBH (**Figure 9B**), which may be associated with cellular development and autoimmunity. In addition, mannose synthesis is present together at different DBH. Unexpectedly, a discrepancy was observed in the expression of *CcGMP6*, which is solely an artificially truncated transcript and contains only the W2 domain that is associated with transcription initiation factors. Although *CcGMP5* also possessed this domain along with other domains, its expression level is not as high as that of *CcGMP6*. It can be inferred that the presence of other domains pfam00132 or Wbbj (involved in acetyltransferase) affects the expression level of *CcGMP5*.

In *CcKTN2* and *CcKTN4*, all genes were involved in the molecular functions of ATPase activity and organic and heterocyclic compounds (**Figure S2B**). Among them, *CcKTN4* was implicated in the molecular function of microtubule severing, as well as the biological processes of cell development and cytoplasmic microtubule organization. *CcKTN2* was involved in the biological processes of endosomal and vacuolar tissues.

## 4 Discussion

Previous studies were rarely related to biological information of *GMP* and *KTN*. In this paper, a total of 8 *CcGMP* genes and 4 *CcKTN* genes were identified. In expression analysis, *CcGMP8* appeared to have no effect on DBH growth. It was reported that the *FaGMP4* gene expression of strawberries of different varieties was different, and the *FaGMP4* of one variety was low in the fruit development stage, resulting in its low AsA content (Lin et al., 2021). Therefore, we speculated that trees with small DBH had relatively low AsA content.

In addition, it was obvious that the *CcGMP4* gene exhibited a higher level of expression compared to other genes, particularly in different DBH. Following closely is *CcGMP6*, which is an artificially truncated transcript based on *CcGMP5* and contains pfam domain pfam02020. This domain is associated with guanine nucleotide exchange factor (Koonin, 1995), and eIF-2B is conserved across eukaryotes, primarily serving as an exchange factor between GTP and GDP while also acting as a key regulatory factor in mRNA translation (Wortham and Proud, 2015). However, it needs to be further verified whether the plant itself can translate proteins containing this domain. In the interaction with *E. grandis* protein, it was observed that the corresponding homologous protein could be identified. The GO annotation results revealed that this homologous protein possesses guanylate exchange factor activity and transcription factor initiation binding, implying the potential interaction of *CcGMP*6 with other proteins to form complex protein-DNA or protein-protein feedback loops regulating gene expression levels.

The paralogous genes of *CcGMP*4 were *At3G55590.1* and *At2G39770.1*. These two genes belong to the key genes of synthetic GMP, but *At3G55590.1* cannot compensate for the functional defect of *At2G39770.1* (Qin et al., 2008). Thus, The function of the *At3G55599.1* gene remains elusive, and further research is needed to fully understand its role. GMP catalyzed the conversion of D-mannose-1-P to GDP-D-mannose, which is a precursor of AsA synthesis. Studies have shown that there is a close relationship between AsA and non-cellulosic cell wall polysaccharide biosynthesis in plants (Gilbert et al., 2009). However, plants lacking *At2G39770.1* tend to exhibit incomplete cell walls and insufficient cellulose content (Nickle and Meinke, 1998; Lukowitz et al., 2001). It means that *CcGMP4* may increase the content of mannose in stem cell wall and maintain the intact plant cell wall.

In the multi-species evolutionary tree, *CcGMP4* contains the conserved domains CL11394 and Pfam00483 of PFAM, while the genes homologous to *AtCYT* in all species share CL11394. This family containing CL11394 domain has the function of glycosyltransferases (GTs). Most of the GTs that catalyze the formation of carbohydrate in plant cell walls have not been biochemically characterized (Brown et al., 2012), mainly because the low abundance of these enzymes in plants makes them difficult to purify in sufficient quantities for enzymatic characterization (Amos and Mohnen, 2019). This also means that GTs that are homologous to *AtCYT* need to be further studied. In addition, The presence of the pfam00483 domain causes *CcGMP4* to have Nucleotidyltransferase activity (Jensen and Reeves, 1998). Furthermore, The presence of M1P_guanylylT_B_like_N on *CcGMP4* protein also verified its GMP function. This enzyme is extremely important for the integrity of cell walls (Jiang et al., 2008), which also indicates that *CcGMP*4 cannot be ignored in the process of cambium cell differentiation.

In addition to domain information, the prediction results of the *CcGMP*4 promoter suggest that it may have function related to cell wall synthesis. In the cis-element analysis results, *CcGMP*4 has elements related to gibberellin, which can promote cell division in cambium to a certain extent (Jeon et al., 2016).

As the reference gene, the annotation result of *CcGMP7* also has the effect of GMP. Its paralogous gene, *At1G74910.2* (*KJC1*), is homologous to human GMPPA (GMP alpha subunits) and likely interacts with *AtCYT1* protein to positively regulate GMP activity, thereby promoting the synthesis of GDP-D-mannose (Zheng et al., 2021). Among them, the mannose content of *vct1-1* was significantly lower than that of *kjc* mutant (Sawake et al., 2015; Nishigaki et al., 2021), indicating that *CcGMP7* and *CcGMP4* had a mutually reinforcing effect, and *CcGMP4* regulated mannose-synthesis in a large proportion. The GO annotation results for *CcGMP7* and *CcGMP4* differed in that the former lacked binding activity with organic and heterocyclic compounds, which may account for its lower expression level compared to the latter. In the comparison of GMP protein sequences (**Figure S3**), it can be inferred that *CcGMP7* and *CcGMP4* are members of GMPPA and GMPPB (GMP beta subunits), respectively, based on the conserved motif (Sawake et al., 2015). It has been reported that genes belonging to GMPPB possess the ability to synthesize GDP-D-mannose (Yi et al., 2021). In the medium DBH, the expression of *CcGMP1* was higher than that of *CcGMP7*. It is possible that *CcGMP1* gene is related to cell development, or that it contains an important GCD1 (Glucosylation Defective 1) domain (Hill and Struhl, 1988), or that there may be some unknown factors leading to the overexpression of *CcGMP1*.

In addition to the promoting effect of GDP-D-mannose on cell wall synthesis, microtubule also has a certain promoting effect on the accumulation of cell wall polysaccharides (Paredez et al., 2006), which provides an idea for selective breeding. The expression level of *CcKTN2* was higher in medium DBH, but lower in other DBH than that of *CcKTN4*. *CcKTN2* and *CcKTN3* were a pair of segmental duplicated genes, and the paralogous gene of *CcKTN3* was *At2G27600.1* (*AtSKD1*). This gene encodes a SKD1 (Suppressor of K+ Transport Growth Defect1) homolog and involved in multivesicular endosome function (Herberth et al., 2012). In the multi-species evolutionary tree, *At2G27600.1* is relatively close to *CcKTN2* and *CcKTN3*, with a conservative domain VPS4 (Vacuolar protein sorting 4) at the C-terminal, which does not cut off microtubules but rather disassembles protein complexes involved in membrane transport (Walker et al., 1982). TESCRT (Endosomal Sorting Complex Required for Transport) controls cell expansion and development in *A. thaliana*, and associated genes have N-terminal microtubule interaction domains (MIT) (Reyes et al., 2014). Notably, the MIT domain is also present in *CcKTN2* and *CcKTN3* (**Figure 6B**). In GO annotation, both *CcKTN2* and *CcKTN3* had ATPase activity, endosome organization and vacuole organization function. They have strong interaction in protein interaction prediction. In the vascular cambium, both the fusiform and ray initials cells are highly vacuolized and both have genes associated with cell wall synthesis (Goue et al., 2008). We hypothesize that *CcKTN2* and *CcKTN3* may regulate the fusion of multivesicular bodies into vacuoles in medium DBH, thereby facilitating material transport and metabolism while maintaining vacuolar integrity (Shahriari et al., 2010). In addition, among the cis-elements of *CcKTN2* and *CcKTN3* promoters, only *CcKTN2* has a 60K protein, which is reported to interact with ESCRT and regulate its localization and function in the cell, thus affecting plant cell division and development (Katsiarimpa et al., 2011). It is concluded that *CcKTN2* plays an important role in plant cell development, but its overexpression level at medium DBH needs to be verified.

*CcKTN4* is a gene of notable significance, exhibiting higher expression levels than *CcKTN2* in both small and large DBH trees. The paralogous gene of *CcKTN4* was *At1G803501*, which primarily encodes the p60 subunit and participates in Katanin, a microtubule-severing enzyme (Burk et al., 2001). *CcKTN4* holds the domain SpoVK (stage V sporulation protein K), which plays an important role in spore formation and division of bacteria (Fan et al., 1992; Hilbert and Piggot, 2004). However, the domains of bacteria and eukaryotes are different. There are some similarities between SpoVK and the ATPase in the AAA+ protein family. SpoVK forms a complex with other proteins, and this complex may be similar to the ATPase complex in the AAA+ protein family, which also plays a role in spore formation in bacteria (Eichenberger et al., 2001; Elsholz et al., 2017). The AAA+ ATPases are enzymes containing a P-loop NTPase domain, and function as molecular chaperones (Iyer et al., 2004). P-loop NTPase domains typically exhibit two conserved sequence characteristics, namely motif GxxxxGK[ST] (Walker A) and hhhh[DE] (Walker B) (Walker et al., 1982), both of which are conserved in the *KTN* genes (**Figure S4**). This domain is ubiquitous in bacteria and eukaryotes (Koonin et al., 2000), and its role in plants is very extensive, involving many biological processes in plants, including cell division (Yu et al., 2016), apoptosis (Lelpe et al., 2004), cellular immunity (Bonardi et al., 2011), etc. Similarly, Katanin also plays a role in cell division in plants (Panteris et al., 2011) and can even promote the elongation of plant cells (Chen et al., 2022). It can be seen that the increase of *CcKTN*4 expression with DBH may be precisely because of the large number of cells and continuous cell division.

In the field of wood property research, genes associated with cellulose synthesis are a crucial research topic. Previous studies have demonstrated that *cyt* and *ktn* mutants exhibit specific effects on cellulose production (Li et al., 2014). This study highlights the significance of *CcGMP4*&*CcKTN4* as a key gene influencing eucalyptus wood size. *CcGMP4* is capable of regulating mannose synthesis, thereby indirectly promoting cell wall synthesis, while *CcKTN*4 can influence the arrangement of cortical microtubules and thus indirectly regulate cell wall synthesis. These findings suggest that *CcGMP4*&*CcKTN4* plays a crucial role in governing growth and wood quality. Therefore, *CcGMP4*&*CcKTN4* can be used for gene knockout or overexpression to further explore the mechanism of wood formation, or through gene editing technology to achieve regulation and optimization of wood formation process.

## Data availability statement

The original contributions presented in the study are included in the article/**Supplementary Material**. Further inquiries can be directed to the corresponding author.

## Author contributions

CW: Conceptualization, Data curation, Investigation, Project administration, Writing – original draft. JL: Methodology, Project administration, Writing – review & editing. WH: Software, Visualization, Writing – review & editing. AH: Investigation, Writing – review & editing. WL: Data curation, Writing – review & editing. YL: Investigation, Resources, Writing – review & editing. YO: Conceptualization, Methodology, Writing – review & editing.
