# Supplementary material for: Genome-wide identification and expression analysis of GDP-D-mannose pyrophosphorylase and KATANIN in Corymbia citriodora
Source: Front Plant Sci. 2023 Dec 18;14:1308354. doi: 10.3389/fpls.2023.1308354 (PMC10766700; doi:10.3389/fpls.2023.1308354)
Supplement: Supplementary file 1 [file DataSheet_1.docx]

Additional Data1. Nucleotide sequences of GMP&KTN genes in *C. citriodora*.

>CcKTN2

MYSNFKEQAIEFVKQAVHEDNAGNYAKAFPLYMNALEYFKTHLKYEKNPKIREAITQKFTEYLRRAEEIRAVLDEGGPGPASNGDAAVATRPKTKPKDGSGGGDGDDAEKEKLRAGLNSAIIREKPNVKWNDVAGLESAKQALQEAVILPVKFPQFFTGKRRPWRAFLLYGPPGTGKSYLAKAVATEADSTFFSVSSSDLVSKWMGESEKLVSSLFQMARESAPSIIFIDEIDSLCGQRGEGNESEASRRIKTELLVQMQGVGHNDEKVLVLAATNTPYALDQAIRRRFDKRIYIPLPDMKARQHMFKVHLGDTPHNLSESDFESLARKTEGFSGSDIAVCVKDVLFEPVRKTQDAMFFIKTSNGMWMPCGPKQPGAVQISMQELAAQGQGSQILPPPISKTDFDKVLARQRPTVSKSDLEVHERFTKEFGEEG

>CcKTN1

MAKSEEEKACWRKEMDEKLNRLHSLLFGADLALDRRDFSTAQILSLRLLGFLDSQTQTDVDAAFIRPIRREASAKLDAARRALTPDSDRRAFQLADRAPGSVFGMKGGIDMEKLKQSTYFRALLQHSKGDSKPMDDQSDKKDNLDKTAKNLKQAKLSSLYGNNLYRQSNAPCKNSPSSRSNNSEDCVILDKPHSIYSHPKSTILSSFPKTEEERFHGNALGAKRVHMEISSPGNNIGKLPSNDEQSNGDAAGNGFVTARAKLEMEARQRHGLAGSPSASVSPQSDSTGRGYGGRSYGSSRRGVRGNFIPPIRSNGNSMGNVTSRVAGGKSDDGIDDSTKRCLEMLCGPDGELPEKLRNLEPRLIEHISNEIMDRDPNVRWDDIAGLDHAKKCVTEMVIWPLLRPDIFKGCRSPGRGLLLFGPPGTGKTMIGKAIAGEAKATFFYISASSLTSKWIGEGEKLVRALFGVAHCRQPAVIFVDEIDSLLSQRKSEGEHESSRRLKTQFLIEMEGFDSGNEQILLIGATNRPQELDEAARRRLTKRLYIPLPSSEARAWIVRNLLEKDGLFKLSSEDINIICNLTKGYSGSDMKNLVKDASMGPLREALRQGTEITKLRKEDMRPVTLQDFEDALQEVRPSVSMNELGIYEEWNKQFGSLSL

>CcKTN3

MYSNFKEQAIDYARQAVQEDNAGNYAKAFALYMNALEYFKTHLKYEKNPKIREAITQKFTEYLRRAEEIRSVLDDGSGGGSSGGGPAAASRPNAKPKDGGEGGDDAEKEKLRAGLNSAIVREKPNVKWDDVAGLEGAKQALQEAVILPVKFPQFFTGKRRPWRAFLLYGPPGTGKSYLAKAVATEADSTFFSVSSSDLVSKWMGESEKLVSNLFQMARESAPSIIFIDEIDSLGGQRGEGNESEASRRIKTEILVQMQGVGNNDEKVLVLAATNTPYSLDQAIRRRFDKRIYIPLPDLKARQHMFKVHLGDTPHNLAESDFESLAQRTEGFSGSDISVCVKDVLFEPVRKTQDAMFFIKTSNGMWMPCGPKQEGAVQISMQELEAEGQAAKIIPPPISRTDFNKVLARQRPTVSKADLEVHERFTREFGEEG

>CcKTN4

MVGSNVAGLQDHLKLAREYALEGLYDTSIIFFDGAIAQINKHLNTVDDPLIRAKWMNVKKALSEEAEVVKQLDAERRAFKETPSGRRPSSPPINTKSSFVFQPLDEYPTSSGAPTMDDPDVWRPPSRDPTGRRPARAGQVGMRKSPQDGTWARGATARAGASARGAKAGGSSKSNTGARASSIGKKGTGSGKSAKADSGNGDAEDGKSKKGQYEGPDQDLAAMLERDVLETTPGVRWDDVAGLSEAKRLLEEAVVLPLWMPEYFQGIRRPWKGVLMFGPPGTGKTLLAKAVATECGTTFFNVSSATLASKWRGESERMVRCLFDLARAYAPSTIFIDEIDSLCNARGASGEHESSRRVKSELLVQIDGVNNSSTNEDGTRKIVMVLAATNFPWDIDEALRRRLEKRIYIPLPNFESRKELIRINLKTVEVATDVNIDEVARRTEGYSGDDLTNVCRDASMNGMRRKIAGKTRDEIKNMSKDEISKDPVAMCDFDEALVKVQRSVSAADIEKHEKWFGEFGSA

>CcGMP7

MGSAAEERVVAVIMVGGPTKGTRFRPLSLNIPKPLFPLAGQPMVHHPISACKRIPNLAQIYLIGFYEEREFALYVSSISNELRVPVRYLKEDKPHGSAGGLYNFRDLIMEDSPSHIFLLNCDVCCSFPLPEMLEAHRRYGGMGTLLVIKVSAELASQFGELIADPSTKELLHYTEKPETFVSDLINCGVYIFTPDIFSAIRNVSTQRKDRANLKRVSSFEALQPATRNLPTDFVRLDQDILSPLAGKRQLYTYETMDFWEQLKTPGMSLKCSGLYLAQFRFTSPHLLASGDGAKVATIIGDVYIHPSAKVHPTAKIGPNVSISANARVGAGARLMNCIILDDVEIKENAVVTHSIIGWKSSIGKWSRAQGGGDYNAKLGITILGEAVTVEDEVVVINSIVLPNKTMNISVQEEIIL

>CcGMP8

MTAALKKYEAEYNVKIEFSVETEPLGTAGPLKLAEKILGKDDAPFFVLNSDVICDYPFKELADFHKAHGEEGTIVVTKVEEPSKYGVVVHKPNHPSRIDRFVEKPIEFVGNRINAGIYILNPSVLKRIDLRPTSIEQETFPSMTKDGQLHSFDLEGFWMDVGQPKDFLSGTCLYLSSLTKKNSKLLTPSTESFVHGGNVLIDPSAKIGQNCKIGPNVVIGPNVVIGDGVRLQRCVLLSASKVKEHAWIKSTIVGWNSVVGRWARLENVTVLGDDVTIGDEIYVNGGSVLPHKSIKANVEVPAIIM

>CcGMP4

MKALILVGGFGTRLRPLTLSVPKPLVDFANKPMILHQIEALKAIGVSEVVLAINYQPEIMLNFLKDFEAKLGIKITCSQETEPLGTAGPLALARDKLLDDSGEPFFVLNSDVISEYPLKEMIEFHKAHGGEASIMVTKVDEPSKYGVVVMEESTGRVEKFVEKPKLFVGNKINAGIYLLNPSVLDRIQLRPTSIEKEVFPKIAAENQLFAMVLPGFWMDIGQPRDYITGLRLYLDSIRKKSSEKLATGPHIVGNVLVHETAKIGEGCLIGPDVALGPGCVVESGVRLSRCTVMRGVRIKKHACVSGSIIGWHSTVGQWARVENMTILGEDVHVGDEIYSNGGVVLPHKEIKASILKPEIVM

>CcGMP5

MAPKRIRARAVSDDPEDLARVPLQAVLLADSFTTRFRPITLERPKVLLPLVNLPMIEYTLAWLESAGVEEVFVFCCAHAKQVMSYLENSQWKSLPKFSVTTIESHNSISAGDALRLIYERNVIHGDFVLISGDTLSNMSLTRALQEHKERKKKDSNAIMTMVVQRSKPSPITQQSRLGTDGLFMAIDPTTKQLLYYEENTNHSKGTLSIDKMLLVDNTSITLHNDIQDCYIDICSLEVLSLFTDNFDYQHLRRHFVKGLLIDDIMGYKIFTHEIHSSYAARVDNYRGYDTISKDIIQRWTYPLVPDVLSLGNSATKLERQGMYRSSEIGQSRSAQIGPHTVIGTGTTIGSNTKITNSVVGKGCTIGSNAVIEGSYVWDNVIIEDECELRHAIVCDGVVMKSRSVLKPGVVLSFKVVIGQNFVVPPYSKVSLLKQPTKQDSDEELEYADNNSDTLDSAATGTMDKLNGDPLLEFSEMEHHVSSELGYGGVGYIWSLCEGSHEEEWRHSVAPIPAEKLAEITQVIEDDLELVSQDSNAPASSGELKPDTQINAEDDAVYFEKEVEATFLRAVNENVEVDHVILEVNSLRLSYNMQAADCAGAIFHSMMKLALDAPHSMQNELLRNVHNTVNAWQKLLKSYLPGKDEEIEVILKFEEICLESVKELSPLFTQILHLLYDKDIITEEAIEDWESEKKDADEADRVFVKQAEIFLQWLREAPEEDDEEDGE

>CcGMP6

SRSVLKPGVVLSFKVVIGQNFVVPPYSKVSLLKQPTKQDSDEELEYADNNSDTLDSATGTMDKLNGDPLLEFSEMEHHVSSELGYGGVGYIWSLCEGSHEEEWRHSVAPIPAEKLAEITQVIEDDLELVSQDSNAPASSGELKPDTQINAEDDAVYFEKEVEATFLRAVNENVEVDHVILEVNSLRLSYNMQAADCAGAIFHSMMKLALDAPHSMQNELLRNVHNTVNAWQKLLKSYLPGKDEEIEVILKFEEICLESVKELSPLFTQILHLLYDKDIITEEAIEDWESEKKDADEADRVFVKQAEIFLQWLREAPEEDDEEDGE

>CcGMP2

MENTGEMEKVVAVIMVGGPTKGTRFRPLSFNTPKPLFPLAGQPMVHHPISACKRIPNLAQIFLIGFYDEKEFTLYVSSISNELKIPVRYLKEDKPHGSAGGLYHFRNLIMEDSPSHIFLLNCDVCCNFPLPDMLDAHKRYGGMGTMLVVKVSAESANQFGTLVSDPVTNELLHYTEKPETFVSDLINCGVYIFTPDIFSAIEDVSNDREDRVTANLRRLSSLDISTSTRTNIPTDFVRLDQDILSPLSGKKQLYTYETMDYWEQIKTPGLSLKCSGLYLAQFRLTSPHLLASGDGKRSATVVGDVYVHPSAKVHPTAKIGPNVSISANVRVGPGVRLICCIILDDVELEDNAVVINSIVGWKSSLGRWSRVQADGDYNSKLGITILGESVVVEQEVVIVNSIVLPNKILNMSVQDEIIL

>CcGMP3

TLVSDPVTNELLHYTEKPETFVSDLINCGVYIFTPDIFSAIEDVSNDREDRANLRRLSSLDISTSTRTNIPTDFVRLDQDILSPLSGKKQLYTYETMDYWEQIKTPGLSLKCSGLYLAQFRLTSPHLLASGDGKRSATVVGDVYVHPSAKVHPTAKIGPNVSISANVRVGPGVRLICCIILDDVELEDNAVVINSIVGWKSSLGRWSRVQADGDYNSKLGITILGESVVVEQEVVIVNSIVLPNKILNMSVQDEIIL

>CcGMP1

MMDFQVVVLAGGFSKKLVPLVSKEVPKALLPVANRPVLSYVLELLEQSNLKDLIVVVEGEDAALLVGGWISGAFVDRLHVEVAAVPEDIGTAGALRAIAHHLTAKDILVVSGDLVSDVPPGAVAAAHRRHNAVVTAMLCSAPVSGPAESGSSVVKDKTKKAGRYNLIGLDHSKQFLLYIATGAELEKDLRIQKSILRAVGEMEIRADLIDAHLYAFKRTVLQEILDQKDTFQSLKEDLLPYLVRSQLKSEILFNGAPQREENANEKVTSQNNQVLLSQILSNSSRPNFHELHELGSYGSASARRTHKCCVYIASKSKYCTRLNSIQAFSDINRDVVGEVNHLSGYSFSSHHNIINPSVKLGSKTTVGPHCMLGEGSQMGDKCSIKRSVIGRHCRIGSNVKIFNSIVMNHVTIGDGCSIHGSVICSNVQLQERVVLKDCQVGAGFVVIAGGEYKGESLAKKEK


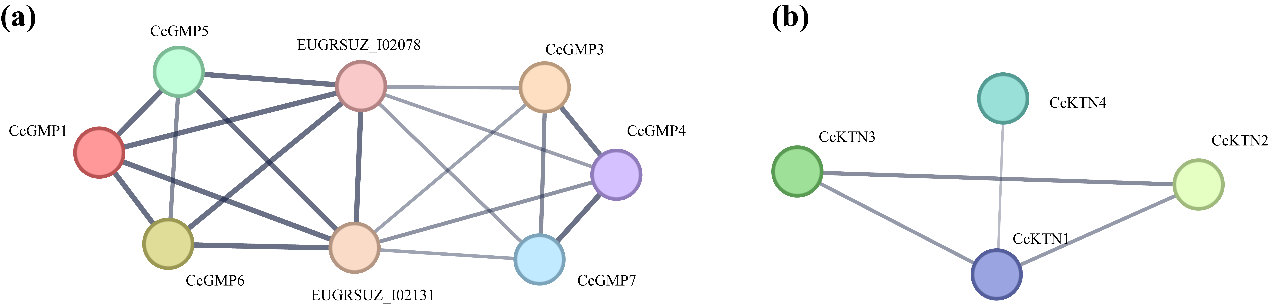


Figure S1. **Putative interactions between *CcGMP*&*CcKTN* proteins**. (a) Prediction of the protein interaction network among *CcGMP*s and additional homology genes. (b) Prediction of the protein interaction network among *CcKTN*s.


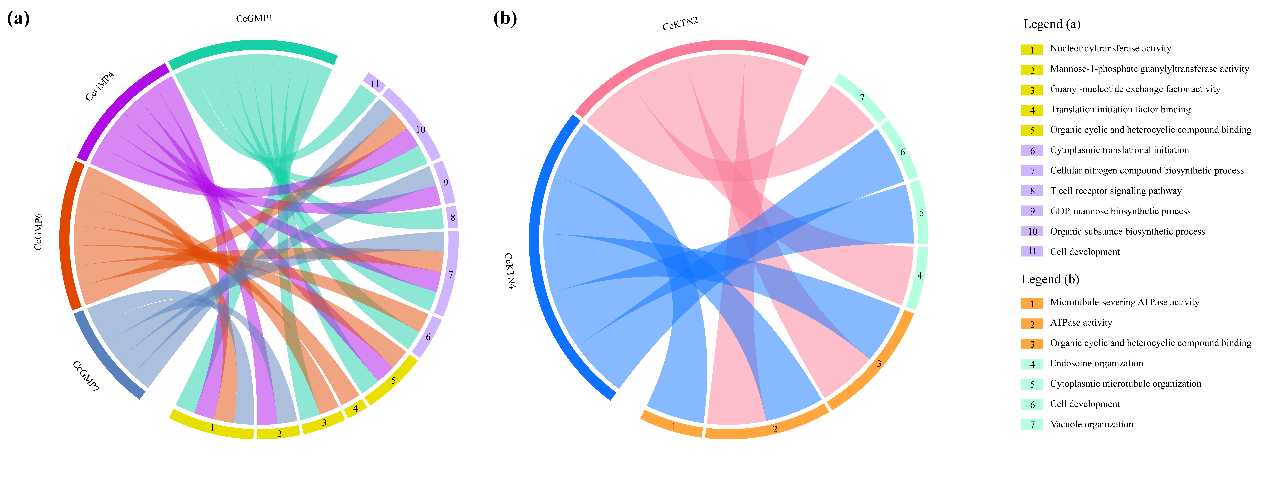


Figure S2. **GO annotation results of reference genes and differentially expressed genes**. (a) GO annotation results of 4 *CcGMP* genes. (b) GO annotation results of 2 *CcKTN* genes. There is at most one annotation for each gene. In Legend (a), 1-5 belongs to molecular functions, 6-11 to biological processes, and the same applies for legend (b) 1-3 and 4-7.


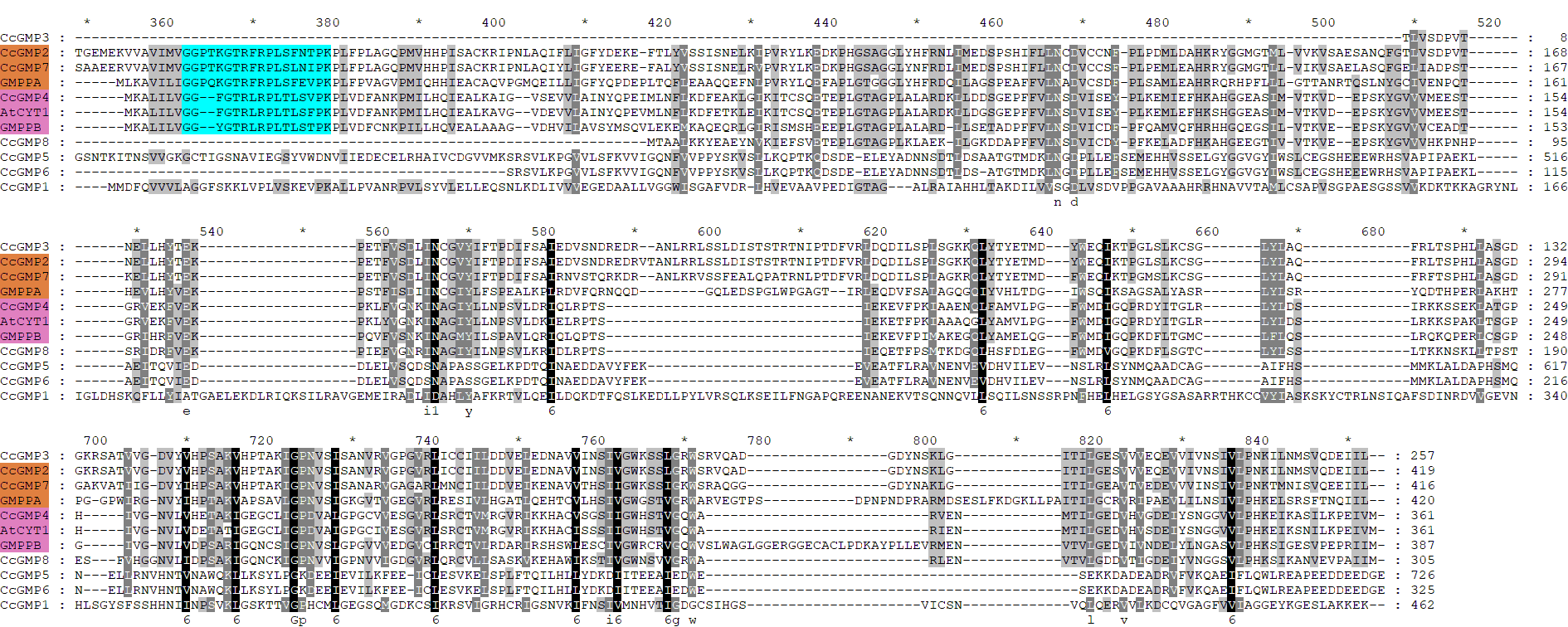


Figure S3. **Alignments of the deduced amino acid sequences of CcGMPs in *C. citriodora****.* The conserved motif that are critical for the identification of GMPs are marked by the shade of cyan. The GMPPA and GMPPB type is indicated by an orange shade and pink shade.


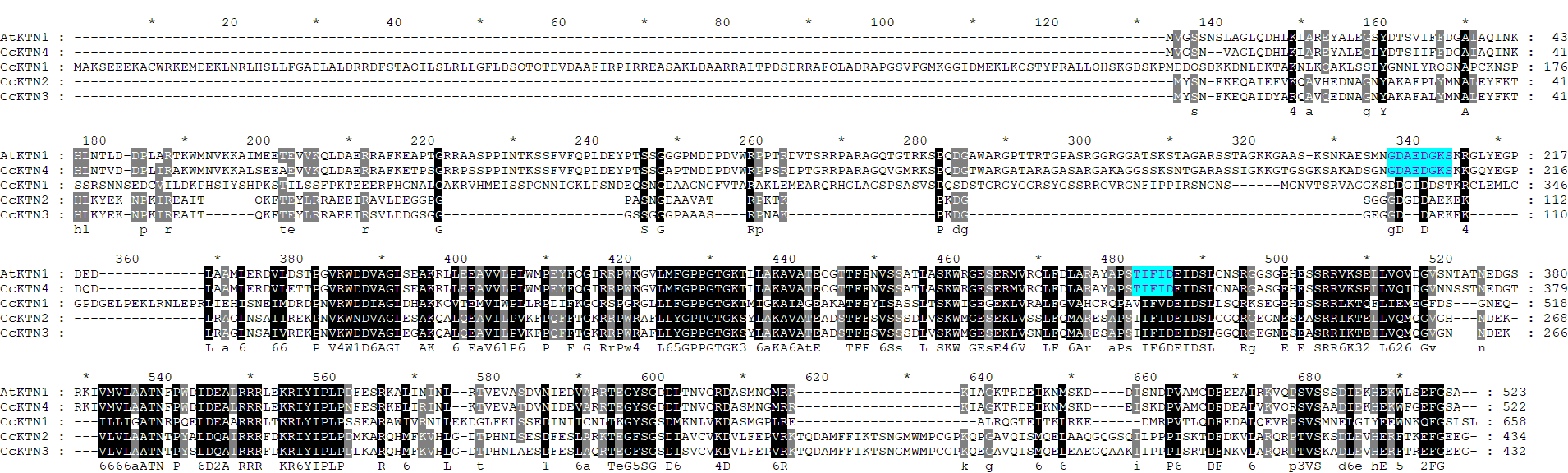


Figure S4. **Alignments of the deduced amino acid sequences of CcKTNs in *C. citriodora****.* The GxxxxGK[ST] and hhhh[DE] motif that are critical for the function of GMPs are marked by the shade of cyan.
